# Effect of diet on the gut mycobiome and potential implications in inflammatory bowel disease

**DOI:** 10.1080/19490976.2024.2399360

**Published:** 2024-09-17

**Authors:** J. Buttar, E. Kon, A. Lee, G. Kaur, G. Lunken

**Affiliations:** a Department of Medicine, University of British Columbia, Vancouver, Canada; b Department of Pediatrics, University of British Columbia, Vancouver, Canada; c BC Children’s Hospital Research Institute, Vancouver, Canada; d Faculty of Land and Food Systems, University of British Columbia, Vancouver, Canada

**Keywords:** Gut, microbiome, mycobiome, diet, dietary modulation, inflammatory bowel disease

## Abstract

The gut microbiome is a complex, unique entity implicated in the prevention, pathogenesis, and progression of common gastrointestinal diseases. While largely dominated by bacterial populations, advanced sequencing techniques have identified co-inhabiting fungal communities, collectively referred to as the mycobiome. Early studies identified that gut inflammation is associated with altered microbial composition, known as gut dysbiosis. Altered microbial profiles are implicated in various pathological diseases, such as inflammatory bowel disease (IBD), though their role as a cause or consequence of systemic inflammation remains the subject of ongoing research. Diet plays a crucial role in the prevention and management of various diseases and is considered to be an essential regulator of systemic inflammation. This review compiles current literature on the impact of dietary modulation on the mycobiome, showing that dietary changes can alter the fungal architecture of the gut. Further research is required to understand the impact of diet on gut fungi, including the metabolic pathways and enzymes involved in fungal fermentation. Additionally, investigating whether dietary modulation of the gut mycobiome could be utilized as a therapy in IBD is essential.

## Introduction

The human gut is inhabited by over 100 trillion microorganisms including bacteria, archaea, viruses, and fungi. Collectively, these are referred to as the gut microbiome. Colonization begins at birth and expands in early life due to increased exposures through diet, travel, and illness.^1,2^ Through adulthood, the microbiome profile stabilizes but remains vulnerable to external stressors. The advent of non-culture-based techniques such as PCR and next-generation sequencing has helped identify the diverse suite of inhabitants of the microbiome. Bacteria comprise 99% of the gut microbiota, largely divided in to Bacteroidetes and Firmicutes phyla.^3^ Within the Bacteroidetes phylum, *Bacteroides* and *Prevotella* genera are prevalent; within the Firmicutes phylum, *Lactobacillus*, *Bacillus*, *Clostridium*, *Enterococcus* and *Ruminococcus* genera are prevalent.^4^ Fungal communities colonizing the gut, collectively known as the gut mycobiome, comprise only 0.01–0.1% of its overall diversity.^5^ Archaea inhabit the gut largely as hydrogenotrophs, serving to decrease the partial pressure of the colon through reduction of carbon dioxide into methane.^6^ Within hydrogenotrophs, *Methanobrevibacter smithii* and *Methanosphaera stadtmanae* are the most prevalent species in the human gut.^7^ Other archaeal phyla such as Crenarchaea and Haloarchaea have been isolated, however, at much lower abundances and are felt to be transient colonizers.^8,9^ Viruses inhabit the gut as either bacteriophages, those that exclusively infect and replicate in bacteria, or eukaryotic viruses, such as *Adenoviridae, Anelloviridae, Astroviridae*, or *Parvoviridae* families.^10^ Given the technical difficulties in accurately extracting single and double strand DNA and RNA, the virome has been historically understudied.^11^ Despite this, the virome has been shown in recent research to play an integral role in the homeostasis of the gut microbiome.^12,13^

The gut microbiome plays important physiological roles in host immunity, harvesting nutrients and strengthening the enteric epithelium.^14–17^ It has been extensively studied as a mediator of health.^18–20^ In comparison with the microbiome profile of healthy controls, the microbiome of patients with various metabolic, neurological, and autoimmune diseases is altered.^21^ One of the most significant influencers of the mycobiome is long-term dietary patterns. Considering the prevalence of the highly processed, low fiber Western diet, its effect on the gut mycobiome remains largely unknown. This review aims to build on current knowledge that analyzes the impact of the mycobiome in gut inflammation, with extension to inflammatory conditions such as inflammatory bowel disease (IBD). In addition, it serves to compile relevant literature to further our understanding of the impact of diet on the mycobiome.

## Methodology

Several authors (JB, EJ, AL and GK) independently searched EMBASE and MEDLINE databases for studies published between June 1, 1947 and July 1, 2024 that evaluated the effect of dietary modulation on the mycobiome. Specific search terms can be found in Appendix A. The authors independently reviewed bibliographies of included studies and review articles to identify further citations not captured by the electronic search. In order for a citation to be included, it had to be a full-text original manuscript, published in English, which directly investigated the impact of dietary modulation on the mycobiome. Dietary modulation was defined as an alteration in the composition of subjects’ oral intake. Mycobiome was defined as the fungal constituents of the gut flora. A total of 9285 full text articles were populated in the initial search. After initial review of the titles and removal of duplicate articles, 242 articles underwent abstract review. Of these, 30 articles underwent full review. A total of 15 articles were included in this review.

## Inflammatory bowel disease

IBD is a class of diseases including Crohn’s disease (CD) and ulcerative colitis (UC), both of which are characterized by a chronic, relapsing-remitting course, with significant patient morbidity and health care burden. IBD is thought to arise from a combination of over-reactive T-cell mediated responses, genetic predisposition, environmental triggers and luminal microbiota.^22^ CD involves all mucosal layers, can be found in any area of the gastrointestinal (GI) tract and is subcategorized into penetrating or stricturing phenotypes.^23^ UC involves the rectum and extends proximally, involving the mucosa and submucosa. In Canada, the prevalence of IBD has increased in the last decade, from 270,000 Canadians in 2018 to 322,600 Canadians in 2023; with numbers estimated to reach 470,000 by 2035. On a global scale, prevalence of IBD has increased 47%, from 3.32 million cases in 1990 to 4.90 million in 2010.^24,25^ The increasing prevalence of IBD has spurred basic science and clinical research endeavors for effective short and long-term therapies, with the unifying goal of decreasing hospitalization and improving patient quality of life.

Pharmacologic therapy in IBD is two-pronged, aiming to either induce or maintain remission. Anti-inflammatory agents, including corticosteroids and 5-aminosalicylic acid, can be administered through various modalities and are most effective in timely induction of remission for patients with active flare.^26^ Immunomodulators and biologic agents, contrastingly, have a slower onset of action but can alter disease trajectory by achieving long-term remission maintenance.^27^ Biologic agents are protein-based molecules that block pro-inflammatory cytokines.^27^ They were initially reserved for patients refractory to immunomodulators as they carry a significant cost to the healthcare system.^28^ In recent years, biologic agents have become the cornerstone of IBD management, supported by evidence demonstrating better long-term prognosis and increased efficacy when introduced earlier in the course of IBD.^29,30^ Further, biologic therapies have become more cost-effective in light of bio-originator compounds exhausting their patency, enabling the advent of bio-similar molecules.^28^ Four different classes of biologic agents are currently approved by Health Canada: anti-TNF agents, anti-integrin agents, anti-interleukin 12/23 IgG1 kappa agents and sphingosine-1-phosphate (S1P) inhibitors.^31^ Janus Kinase inhibitors (JAK), commonly referred to as small molecule inhibitors, remain on the horizon with promising clinical response and superior patient convenience by offering oral administration.^32^ The advent of biologic therapies has led to fewer IBD-related hospitalizations and corrective abdominal surgeries, however, the overall morbidity associated with IBD remains high.^33^

Non-pharmacologic therapies, such as dietary modulation, have significant benefits as they are cost effective and empowering to patients. It is supported in other areas of medicine, such as cardiovascular health, as an adjunct to conventional, pharmacologic therapies to reduce risk and progression of atherosclerosis.^34^ However, gaps in our understanding remain regarding dietary effect on IBD progression despite its accepted role in its pathogenesis.^35,36^ Current guidelines are unable to define an “IBD diet” that can promote remission in adult patients with active disease.^37,38^ To rectify this, we must bolster our understanding of the impact of diet on the human gut, starting with a better understanding of the relationship between diet and the gut microbiome and their role in inflammation.

## Fungi in the gut

Fungi are unicellular or multicellular, heterotrophic eukaryotes with an impressive ability to decompose and harvest otherwise unavailable nutrients.^39^ Influenced by historical studies associating *Candida* as a facultative pathogen, early research focused on the role of fungi as a potential detriment to gut health.^40,41^ With the advent of works such as the Human Microbiome Project, fungal species have gained momentum as a necessary component of the microbiome with both commensal and mutualistic relationships.^5,42^

To delineate which fungal species are autochthonous and contribute toward gut health, the mycobiome is categorized into resident and nonresident species. Resident species have intrinsic ability to grow in the anoxic mammalian gut environment, known for its variable pH and increased physiologic temperature of 37°C. This subgroup includes genera within the Ascomycota, Basidiomycota, and Zygomycota phyla, such as *Candida, Malassezia* and *Cladosporium*.^5,43^ In contrast, nonresident species are isolated in the gut using non-culture techniques, and are not currently felt to contribute to gut physiology. However, this is likely an overly simplistic way of characterizing fungi. Common nonresident species include *Saccharomyces, Aspergillus*, and *Penicillium*, among many others. ^43^ Nonresident species are often the product of external influences such as short-term dietary exposures or environmental triggers. This sub-group makes up two-thirds of isolated fungal species.^44^

### Candida in the gut

The most frequently reported genus among resident fungi is *Candida*, including *C.albicans, C.tropicalis, C.parapsilosis*, and *C.glabrata* species.^45^ *C.albicans*, a dimorphic fungus, is most well known as an opportunistic pathogen of the gut, oral mucosa, vagina and blood.^46^ It is responsible for millions of cases of vaginal and oral infections every year, conferring significant mortality when infecting an immunocompromised host.^47^ However, *C.albicans* also demonstrates an impressive commitment to gut commensalism when it remains in its yeast form. In particular, *C.albicans* undergoes a “gastrointestinal induced transition” when exposed to the mammalian gut, thought to be in part due to upregulation of the *WOR1* transcription factor, that results in phenotypic changes more suitable for nutrient uptake of short chain fatty acids (SCFA) and N-acetylglucosamine.^48^ What dictates the morphologic shift that drives *C.albicans* into an invasive pathogen is not fully understood, with preliminary research highlighting a novel, regulatory role of host epithelial cells.^49^

### Malassezia in the gut

Known largely as a colonizer and pathogen of the skin, the *Malassezia* genus is also the second most prevalent fungal species within the gut.^43,50^ Among these, *M.globosa, M.restricta, M.sympodalis*, and *M.pachydermatis* are the most prevalent species in the gut.^51^ Nearly all species of *Malassezia* do not synthesize fatty acids and instead rely on external sources for lipids, explaining their proliferation on sebum-rich producing areas of the human skin.^52^ Within the gut, lipids are available from bile salts, synthesized from bile acids by hepatocytes.^53^ It remains poorly understood how *Malassezia* colonize the human gut, with competing hypotheses including indirect exposure from human skin or because of breast milk ingestion during infancy.^53^ Regardless of the mechanism, *Malassezia* is a predominant player of fungal colonization, with recent studies implicating it in gut symbiosis.^43,54^ In certain circumstances, however, *Malassezia* has pathogenic potential, inducing detrimental, aberrant immune reactions.^55^ On the skin, it has been linked to the pathogenesis of seborrheic dermatitis and pityriasis versicolor.^56^ *Malassezia* has been found in greater relative abundance in pancreatic ductal adenocarcinoma tissue compared to gut, implicated in oncogenesis through stimulation of the pattern recognition receptor mannose binding lectin (MBL).^57^ *Malassezia* is found at greater relative proportions in patients with IBD, compared to healthy controls, and is thought to contribute toward mucositis.^58^

### Saccharomyces in the gut

The *Saccharomyces* genus is considered a nonresident fungus of the human gut, with the most predominant species being *Saccharomyces cerevisiae*. *S.cerevisiae* is commonly known as baker’s yeast and one of the most well-studied single-celled organisms. It is instrumental in leavening of bread, brewing of beer and commonly used as a food additive. Its role in the homeostasis of the gut microbiome is often overlooked as it was historically thought to be a transient colonizer and consequence of dietary habits. However, recent research has shown *S.cerevisiae* can indeed thrive in the gut, metabolizing the highly glycosylated protein known as mucin.^59^ A strain of *S.cerevisiae*, known as S.*cerevisiae* var. *boulardii*, is a well-known probiotic yeast species known to help resolve various GI diseases, including traveler’s diarrhea and antibiotic-associated diarrhea.^60,61^ The use of *S.cerevisiae* var. *boulardii* in the treatment of CD has low-grade evidence in consensus guidelines, after small pilot studies demonstrated decreased colonic permeability in CD patients.^62,63^

## Impact of the gut mycobiome on immune responses

Gut fungi impact host immunity through the innate and adaptive immune system, initiated by the interaction between fungal cell wall components and pattern recognition receptors (PRR) of intestinal immune cells. Phagocytosis of fungi leads to activation of various immune responses, in an effort to control fungal colonization.^64^ When these regulatory mechanisms are dysfunctional or absent, fungal colonization is left unchecked, leading to disturbance of gut microbial architecture and intestinal inflammation.^65^

Through several checkpoints, the innate and adaptive immune system can distinguish between commensal or pathogenic fungi. Given that *C.albicans* can act as either a commensal or pathogenic agent, it is often applied in immunologic analyses to help differentiate these distinct immune responses.^66^ The protein associated molecular patterns (PAMPs) of *C.albicans*, and other fungi, interact with toll-like receptors (TLRs), C-type lectin receptors (CLRs) and NOD-like receptors (NLRs) on intestinal mononuclear phagocytes (MNPs).^67^ As an example, macrophage mannose receptor 1 (MMR), dendritic cell-specific ICAM3-grabbing non-integrin (DC-SIGN), and macrophage-inducible C-type lectin (MINCLE) all recognize mannose-rich structures on the cell wall of certain fungi.^68^ Common C-type lectin receptors that MNPs express are dectin-1 and dectin-2. Binding of dectin-1 to β-glucans of fungi stimulates a conformational change, exposing immunoreceptor tyrosine-based activation motifs (ITAM) on its tail to be phosphorylated by SRC family kinases.^69^ Phosphorylation of ITAMs on dectin-1 allow tandem Src-homology 2 (SH2) domains of spleen tyrosine kinase (Syk) to dock, which activates it to phosphorylate caspase recruitment domain-containing 9 protein (CARD9).^64^ Activation of CARD9 leads to induction of Th-17 cells, which mobilize neutrophils to the intestine.^70^

Binding of dectin-1 to β-glucans on *C.albicans* or *Aspergillus* also promotes activation of bio-active IL-1β from its non-active form, pro-IL-1β through the noncanonical caspase-8 inflammasome.^71^ Importantly, activation of caspase-8 inflammasome does not rely on internalization of fungal material.^71^ As such, dectin-1 can mount an effective and swift response after recognition of external fungal cell wall components through activation of IL-1β. IL-1β is a member of the IL-1 family, known for inducing systemic inflammation. However, it can also function to protect against foreign antigens. In response to fungal recognition, IL-1β stimulates helper T cells, mobilizes neutrophils, and enhances phagocytosis in an effort to improve clearance of fungal pathogens from the gut.^72–75^

When *C.albicans* is seen in its commensal, yeast morphology, its PAMPS responsible for inflammasome and IL-17 pathways are hidden.^67^ When *C.albicans* transitions to its pathogenic, hyphae form, both the inflammasome pathway and IL-17 signaling pathway are activated.^76^ In particular, TLRs secrete cytokines including IL-1β, IL-23, and IL-6, which favor activation of CD4^+^ T cells into the Th17 lineage.^77^ Th17 cells are responsible for secreting IL-17, a cytokine which recruits macrophages, neutrophils and antimicrobial peptides to mount an effective anti-fungal response.^77^ Thus, the unique interaction of PAMPs on pathogenic fungi and PRRs on host dendritic cells dictate the inflammatory response.

The mycobiome plays an important role in host immunity. The macrophages and monocytes involved in fungal recognition undergo trained immunity after exposure to commensal fungal species such as *S.cerevisiae*, increasing TNF-alpha and IL-6 production in response to further exposure of bacterial or fungal organisms.^78^ Fungal commensals themselves play a direct role in the stabilization of gut homeostasis, with *C.albicans* able to shed its pathogenic strains in times of host stress, protecting it from bacterial and fungal pathogens.^79^ Fungal symbiosis also plays a protective role against colitis-related carcinogenesis. Mice depleted in fungi showed higher rates of colon tumorigenesis after azoxymethane – dextran sodium sulfate (AOM/DSS) administration compared to wild-type mice. When supplemented with exogenous IL-18, rates of tumorigenesis decreased significantly.^80^ As such, fungal symbiosis plays an important role in host immunity in protecting against colitis and colitis-associated cancer through the inflammasome pathway.

## Inter-kingdom relationships

The GI tract hosts an array of microorganisms residing together to form complex and dynamic ecosystems. Recent attention has been paid to bacterial-fungal interactions as they have been implicated in disease.^81^ Bacterial-fungal interactions can be mutually beneficial, however, competition to establish a specific niche or for nutrients may result in an imbalance in bacteria and fungi. There are various mechanisms involved in both mutualistic and antagonistic bacterial-fungal communication. One study demonstrated that SCFA production by bacteria during fiber fermentation carried intrinsically anti-fungal qualities.^82^ Another study postulated that peptidoglycan fragments secreted during bacterial cell wall synthesis directly bind and inactivate *C.albicans*, preventing its transition from a budding yeast to elongated hyphae.^83^ Through these mechanisms, a stable and diverse bacterial gut community regulates its fungal counterparts. During times of intestinal inflammation or prolonged antibiotic use, abundance of bacteria in the gut decreases, rendering it susceptible to invasive fungal species.^55^ This was highlighted in a study involving mice treated with vancomycin which showed disseminated *C.albicans* infection in the absence of bacterial species.^84^ As such, pathogenic fungi are regulated not just by host immunity but also by various commensal bacterial species.

The finite supply of nutritional resources in the gut, particularly metals such as iron, has led to various relationships between fungi and bacteria.^85^ Non-reductive mechanisms of iron extraction involve siderophores, secreted molecules that scavenge iron in the environment and are re-absorbed.^86^ Commensal relationships soon emerged, such as mutant *Escherichia coli* strains that are deficient in siderophore synthesis and adapted to utilize those produced by *Penicillium*, such as ferrichrome and coprogen.^87^ Contrastingly, *Pseudomonas aeruginosa* and *Aspergillus fumigatus* secrete siderophores only they can assimilate, further reducing nutrient supply from their competitors.^88^

Fungi and bacteria interact to optimize their microenvironment. Gut organisms produce an extracellular matrix known as the biofilm to protect against antimicrobial factors.^89^ In vitro studies showed *C.tropicalis*, *E.coli* and *Serratia marcescens* work symbiotically to create a significantly thicker biofilm than they are capable of producing alone.^89,90^ Scanning electron microscopy revealed the intimate interaction of these three organisms, with *S.marcescens* using fimbriae to connect *E.coli* and *C.tropicalis* together.^91^

## Gut mycobiome in IBD

The microbiome profile in IBD patients is known to be altered, with historical studies largely focusing on the relative imbalance of bacteria.^92^ IBD patients have reduced bacterial biodiversity, with a relative decrease in bacteria from the Firmicutes phylum and a relative increase in bacteria from the Proteobacterium phylum.^93^ The mycobiome in IBD patients comprises largely of fungi from Ascomycota and Basidiomycota phyla, with the most prevalent genera including *Saccharomyces, Debaryomyces, Penicillium, Aspergillus*, and *Candida*.^94,95^

IBD involves a heterogenous collection of disease phenotypes. The phenotype of disease, and extent of inflammation, is associated with a unique mycobiome profile. In CD, the mycobiome profile differs based on disease involving the ileum compared to disease exclusively in the colon. In particular, ileum-sparing CD showed higher rates of *Candida* and *Debaryomyces*, while *Aspergillus* and *Pichia* were in higher abundance in ileal-involved CD.^95^ During a disease flare, the ratio of Basidiomycota to Ascomycota increases compared to controls.^94,95^ On a species level, CD patients in an active flare showed greater relative abundance of *Candida* species, *Gibberella moniliformis*, *Alternaria brassicicola* and *Cryptococcus neoformans*.^96^ Variations of the mycobiome profile is best highlighted in UC when categorized on extent of disease. Patients with proctitis had the highest relative abundance of *Penicillium*, negatively correlated as extent of disease progressed proximally.^95^ In contrast, *Pichia* was over-represented in patients with left-sided colitis compared to those with proctitis.^95^

As the unique microbiome profile associated with IBD flare has become more validated, the concept of fungal pathobionts contributing toward a pro-inflammatory state has emerged, starting with *C.albicans*.^90^ The abundance of *C.albicans* is increased in the gut of IBD patients compared to healthy controls.^97^ In its hyphal form, *C.albicans* upregulates the endothelial converting enzyme 1 (ECE1) gene, leading to greater abundance of the ECE1 protein.^98^ This protein undergoes proteolytic processing to release its active form, known as Candidalysin. Candidalysin is a 31-amino acid cytolytic peptide, known for damaging epithelial cell plasma membranes and stimulating transcription factors that encode pro-inflammatory cytokines.^99^ Candidalysin also damages the function of macrophages, negatively impacting their antifungal capacity.^100^ Interestingly, a cohort of CD patients demonstrated lower levels of IgA-mediated responses specific to Candidalysin compared to healthy controls.^101^ This suggests that highly immunogenic fungal pathogens are not sufficiently controlled in IBD patients and may contribute to its pro-inflammatory state.

As a by-product of fungal profiling in IBD, certain species have been identified as surrogate markers of inflammation. *S.cerevisiae* is found in greater relative abundance in non-inflamed tissue compared to inflamed tissue within the same CD patient.^102^ CD patients who are actively flaring show higher levels of anti-*S.cerevisiae* antibodies (ASCAs).^102^ ASCA have also been utilized in disease forecasting, with higher levels of ASCA found in patients later diagnosed with CD compared to controls.^103^ Additionally, ASCA has been extrapolated as a predictor of disease severity, with patients with positive ASCA IgG having higher rates of surgical interventions compared with CD patients with negative ASCA IgG.^104,105^ Regular screening of CD patients with ASCA has yet to permeate into regular practice in clinical medicine, however it continues to show promise as an adjunctive, noninvasive measure of disease activity and severity.

## Isolating and analyzing the gut mycobiome

More than 390 fungal species have been identified across diverse body sites including the skin, urogenital, respiratory, and GI tracts. However, more than 99% of microbial genes in the gut belong to bacteria, with fungi constituting a minor fraction of the resident microbiota.^106^ Despite low fungal diversity and abundance compared to bacteria, considerable variability exists between individuals, however, several fungal species are shared across individuals suggesting a core gut mycobiota may exist.^107^ Moreover, the composition and function of fungi have been linked in various diseases, including IBD, colorectal cancer and metabolic syndrome.^108^ Influencing factors include mode of delivery, breastfeeding, diet, environmental fungal exposure, and age.^108^ However, microbiome research predominantly concentrates on bacteria, with insufficient attention given to other crucial microbial groups such as fungi. Consequently, microbiota analysis techniques often exhibit bias toward the bacteriome, hindering mycobiome studies due to suboptimal extraction methods, technical challenges, limited fungal databases, and biases in data analysis.

Designing and executing mycobiome-focused studies present several challenges. First, the choice of sample collection method significantly impacts results, favoring direct freezing over preservatives, which can adversely affect fungal species abundance.^5,109^ Another crucial consideration is extraction of fungal DNA, particularly when both fungal and bacterial communities are of interest. Optimal extraction methods are essential to achieve high DNA yields. Challenges arise in mixed sample types, such as stool, where the robust nature of fungal cell walls, consisting of chitin, ß-glucan, mannans and glycoproteins, requires repeated bead beating and enzymatic lysis to ensure adequate cell lysis and optimal extraction of fungal DNA. A review of fungal studies revealed the use of 33 different commercial extraction kits and 13 different customized protocols, indicating a need for standardized protocols.^108^

Several studies have compared the efficacy of different fungal DNA extraction kits.^110–112^ Shaffer et al. evaluated five commercially available kits (NucleoMag Food, Zymo MagBead, PowerSoil, PowerSoil Pro, and MagMax Microbiome) and found that for extracting fungal DNA from stool, the PowerSoil Pro and MagMax Microbiome kit outperformed the others.^111^ Another study comparing various DNA extraction kits (QIAamp DNA Stool, PureLink DNA, ZR Fecal DNA MiniPrep, NucleoSpin, IHMS protocol Q) showed that the noncommercial IHMS protocol Q, recommended by the International Human Microbiome Consortium, and the ZR Fecal DNA kit were the most effective.^112^ Given the typically low fungal biomass in stool samples, it is crucial to assess fungal kit contamination during the DNA extraction process by including water blanks.^112^

Various sequencing techniques can assess fungal composition and functional capacity within a sample. Amplicon sequencing, targeting the ubiquitous fungal rRNA gene locus, specifically the ribosomal small subunit (18S) and the large subunit (26S) regions separated by the internal transcribed spacer (ITS) regions ITS1 and ITS2, is a common approach.^113^ Some studies suggest that ITS fragments promote shorter amplified sequences, however, more curated and rich databases are available often making ITS preferable to 18S or 26S sequencing.^114^ Alternatively, metagenomic shotgun sequencing captures total DNA, including human, bacterial and fungal components. However, due to low fungal DNA concentrations, deeper and more expensive sequencing is required to generated sufficient reads. Additionally, reference databases for annotated fungal sequences are limited compared to bacteria necessitating well-curated databases like UNITE, NCBI RefSeq Targeted Loci, and MaarjAM to ensure reliable fungal taxonomic classification.^115^ A concerted effort to better characterize fungi is crucial to addressing these challenges and advancing mycobiome research.

## Effect of diet on the gut microbiome

Given the extensive diversity within the gut microbiome and its unique, multi-modal effect on human health, mechanisms to optimize the gut microbiome to improve health have become the focus of many emerging research projects.^116,117^ Diet is not only a vector for many microbial constituents within the GI tract but also the primary nutrient source for its inhabitants. As such, the modulation of diet can indirectly propagate pro- or anti-inflammatory effects through changes in microbiota composition and function.^118,119^ To date, the impacts of diet modulation have largely focused on changes in composition and function of bacterial populations. However, given the importance of fungal communities in gut health, further research on the impact of diet modulation on this important subcommunity is warranted.

Dietary modulation is a growing field in the world of non-pharmacologic therapies for many GI diseases, as it empowers patients to play an active role in their treatment plan while also serving as a cost-effective, low-risk alternative to pharmacologic treatments. Dietary modulation encompasses various dietary modifications, ranging from a low fermentable oligosaccharides, disaccharides, monosaccharides and polyols (FODMAP) diet to fiber-enriched foods. These recommendations often stem from prior research linking diet with gut health. Diets rich in tryptophan and fiber have been shown to confer immune states favoring gut health.^75^ Contrastingly, foods containing artificial sweeteners and emulsifiers, found in processed foods, can lead to increased gut permeability and intestinal inflammation.^120,121^ The Western diet is comprised of refined carbohydrates with high fat content, thought to confer lower microbial diversity compared to people in rural countries with greater access to whole foods.^119^ Given that most Westerners do not meet their daily fiber requirements, the impact of a fiber-enriched diet on gut health has become an emerging topic.^122^ The benefits of dietary fiber are multi-faceted and primarily stem from its fermentation into SCFA, particularly butyrate, acetate, and propionate. These SCFA are known to provide energy for colonic epithelial cells while also dampening down inflammatory cascades and aberrant cellular replication processes.^123^

Dietary modification plays an integral role in shaping the composition, function and diversity of the gut microbiome, and its effects are most often seen when applied over a long-time course. Gut microbiota are intrinsically resilient to temporary dietary regimens, with up to 60% of bacterial strains persisting for over 5 years despite intermittent dietary changes.^124^ As such, recommendations for dietary modifications must acknowledge feasibility, cost, and patient adherence, all of which are limiting factors in restrictive diets such as low FODMAP regimens.

## Dietary therapies in IBD

IBD patients often inquire about dietary strategies to manage their GI symptoms. Studies indicate that dietary treatments can serve as adjunct therapies, modulating inflammation and promoting mucosal healing.^125^ The most compelling evidence supports utilizing exclusive enteral nutrition (EEN) in pediatric CD patients. EEN, involving a nutritionally complete liquid diet without food intake for 8–12 weeks, shows efficacy similar to steroids, promoting growth without associated side effects.^126^ Partial enteral nutrition (PEN), allowing some food intake, is better tolerated than EEN, with the Crohn’s disease exclusion diet (CDED) as an example. CDED combines PEN with a diet avoiding or reducing animal/dairy fat/proteins, wheat, and food additives, allowing gradual reintroduction of fruits, vegetables, and legumes to improve food flexibility. Some diets for adult IBD patients, such as the specific carbohydrate diet (SCD), have shown promise in reducing GI symptoms. SCD restricts complex carbohydrates thought to be poorly absorbed by patients with IBD, avoiding grains, starchy vegetables, dairy products, sugars and sweeteners, and certain food additives.^127^ The Mediterranean diet has gained recent attention for being less restrictive yet as effective as the SCD.^128^ This diet emphasizes fruits, vegetables, nuts, fish, whole grains, oily fish, and olive oil known to reduce inflammation, while limiting animal fats/protein and food additives which have pro-inflammation properties.^129^ A Cochrane systematic review on diet for CD remission induction concluded that the evidence is low or very-low quality.^130^ This is possibly due to variable patient response, suggesting a personalized approach is likely more effective. While dietary fibers are generally considered beneficial to health, some IBD patients, especially during active disease, have reduced tolerance to high fiber containing foods.^131,132^ Armstrong et al. demonstrated in active disease patients, that certain fibers, especially oligo-fructose (FOS), triggered pro-inflammatory cytokine IL-1β.^133^ Fermentation of FOS with microbiota from non-IBD or mildly affected patients reduced IL-1β secretion. However, this reduction was absent in microbiota from most IBD patients with moderate or severe disease, indicating insufficient fermentation of FOS by commensal microbiota might lead to abnormal immune responses. Bonazzi et al. recent study demonstrated considerable inter-individual variability in gut microbiota responses to different fibers using an in vitro microbiota modeling system.^134^ Microbiota composition and function varied widely with inulin and psyllium fibers, with some individuals having a fiber-resistant microbiota, while others had a fiber-sensitive microbiota. These studies underscore the necessity for personalized dietary recommendations, especially in managing gut health and inflammation.

## Dietary modulation and its effect on the gut mycobiome

As response to diet, particularly in the context of IBD, is at least partially mediated by the gut microbiome, there is need for a more comprehensive understanding of how various nutrients and food groups contribute to altering specific members of the gut microbiota, extending beyond focusing solely on bacteria.^135^ Many studies exploring the role the gut microbiome plays in mediating the effect of diet on gut health concentrate solely on bacteria. Targeting gut bacteria through diet therapies can lead to shifts in both host and microbial factors.^136^ For instance, a plant-based diet was associated with a shift in gut bacteria toward a more anti-inflammatory profile, characterized by higher abundances of *Faecalibacterium prausnitzii*, *Eubacterium rectale* and *Eubacterium biforme*, all of which are butyrate producers. Conversely, adherence to a meat-based diet was associated with a more pro-inflammatory profile, characterized by *Ruminococcus gnavus* and *Collinsella* species.^137^ Despite these and similar findings, the majority of diet-microbiome studies do not extend their focus beyond bacteria to include other microbes such as fungi. Exploring the impact of diet on microbes outside of bacteria, including fungi, is essential. This broader perspective may enhance our understanding of how diet can target all key members of the microbiota, providing comprehensive insights into their implications on gut health and disease.

### Carbohydrate-rich diets

The digestibility of dietary carbohydrates determines their metabolic fate. Digestible carbohydrates, such as monosaccharides, disaccharides, and starch polysaccharides, are broken down into energy after being digested and absorbed by the body. In contrast, most nondigestible carbohydrates or fibers interact with the gastrointestinal microbiota and undergo fermentation primarily in the colon. This fermentation process produces gas, SCFA, and other metabolites.^138^

Hoffman et al. showed that *Candida* is positively correlated with recent carbohydrate consumption. (Table 1).^139^ Additionally, Pizzo et al. found that high intakes of fructose, glucose, maltose, and sucrose increased the adhesion of *Candida* species (*C.albicans, C.tropicalis*, and *C.krusei*) to epithelial cells.^140^ Moreover, reducing dietary monosaccharides and starch has been shown to decrease chronic *Candida* overgrowth in the intestine.^141^

**Table 1.** Impact of dietary carbohydrate modulation on gut mycobiome in human studies.

Author	Diet	Number of participants	Study design	Microbial Changes
Hoffman et al.^139^	Retrospective diet inventories (long-term vs short-term diet).	96	- Long-term diet assessed using an FFQ and short-term diet assessed using 3-day diet recalls - Fungi characterized by sequencing the ITS1 and 16S rRNA locus. - Clustering method used to identify co-varying groups of dietary components (carbohydrates, protein, amino acids).	- *Candida* positively correlated with intake of carbohydrates and negatively associated with intake of total saturated fatty acids. - *Aspergillus* negatively correlated with intake of SCFA.
Otasevic et al.^141^	Study-specific diet included: avoidance of alcohol, smoking, milk and dairy products, simple sugar-containing foods, cured and fatty meats. Recommended whole grain bread and whole grain pasta, artificial sweeteners, low-fat white meat, fish, seafood, acidophilus drinks and supplements.	120 patients with ICOG (*n* = 80 SG; *n* = 40 CG)	- Pilot study – Adherence to 3 months of diet regime and 10 days of 2 × 500.00 IU nystatin TID in SG - Adherence to 10 days of 2 × 500.00 IU nystatin TID provided in CG - Two mycological control examinations and follow-up post-treatment: first follow-up within 10 days after anti-fungal treatment completion; second follow-up occurs 3 months after the initiation of treatment	- Adherence to study-specific diet led to lower levels of *Candida* compared to patients who were treated with nystatin only
Pareek et al.^142^	Retrospective diet inventories	47 Japanese adults (25 Male, 22 Female) AND 50 Indian adults (27 male, 23 female)	- Comparative study assessing fecal samples from both Japanese and Indian groups - Fungal DNA was extracted and rRNA was amplified via PCR in the ITS1 region, followed by SMRT sequencing - Fungal composition determined by PacBio technology	- A higher proportion of *Candida* and *Prevotella* were found in healthy Indian participants compared to the healthy Japanese population
Tian et al.^143^	HC regimen (65 – 76% carbohydrate) or LC regimen (15 – 25% carbohydrate)	28	- Cross-over study with 3 cycles − 6 days of HC diet, 6-day washout period, followed by six days of LC diet – or vice versa - Fungal rRNA amplified via PCR from purified DNA	- HC regimen: Relative increase in *Pleurotus, Kazachstania, Auricularia, Ustilaginaceae*; Relative decrease in *Ustilaginaceae* - LC regimen: Relative increase in *Ustilaginaceae*; Relative decrease in *Blumeria, Agaricomycetes, Malassezia, Rhizopus, Penicillium*
Shuai et al.^149^	Retrospective diet inventories (long-term habitual diet)	1244	- Taxonomic profiling and comprehensive multi-omics analysis were used to identify ecological links between the fecal metabolome, bacteria, and fungi	- Dairy consumption was negatively associated with *Candida* but was positively associated with *Saccharomyces*
Sun et al.^153^	Retrospective diet inventories (long-term habitual diet)	942 healthy Chinese participants from 6 ethnicities: Han, Zang, Bai, Hani, Dai, and Miao	- Cohort study where shotgun metagenomic sequencing was used to profile fecal mycobiome samples - Dietary habits from one month was collected via a questionnaire. - The participants’ metadata (environmental exposure, bowel habits, anthropometrics, medication) were collected.	- Mushrooms were exclusively correlated with 3 fungal species (*Rhizopus stolonifer, Puccinia Sessilis*, and *Botryozyma* species) - Black tea and pork are significantly correlated with 4 fungal species from the genus *Fusarium* - Ingestion of buttermilk and blueberry tea is correlated with *Tetrapissipora blattae, Sugiyamaella lignohabitans, Kazachstania naganishii*, and others.
Ukhanova et al.^154^	Low-fiber American diet in addition to 0, 1.5, or 3 servings of almond or pistachio nuts	34 (almond study = 18; pistachio study = 16)	- Two separate randomized, controlled cross-over studies of almond and pistachio nuts with 3 × 18-day feeding periods separated by a washout period of at least 2 weeks - Fungal rRNA amplified via PCR and ITS sequences were analyzed	Consumption of almonds and pistachio nuts are negatively associated with abundance of *Candida* and *Penicillium* genera.
David et al.^155^	Plant-based (32% fat, 16% protein) OR Animal-based (69% fat, 30% protein)	10 (6 male, 4 female)	- Cross-over study with a 5 day intervention period and one month washout period - Sequencing of the ITS region mapped to RNA-seq from a reference set of fungal genomes	Animal diets had greater fungal diversity and increased *Lactococcus lactis* and *Neosartorya fischeri*.
Hallen-Adams et al.;^44^ Suhr et al.^50^	Hallen-Adams et al.: Conventional (Western) diet Suhr et al.: Vegetarian diet	Hallen-Adams et al.: 45 participants (21 sampled at one-time point; 24 sampled at two-time points) Suhr et al.: 16 self-identified vegetarians	Hallen-Adams et al.: - Fungal rRNA was amplified via PCR and sequenced at one or two time points using the fungal-specific forward primer ITS 1F and eukaryotic reverse primer TW13. - Clustering method was used for sequence analysis and subjected to BLAST search against GEnBank’s non-redundant nucleotide database Suhr et al.: - Fungal rRNA from 16 fecal samples were amplified via PCR and identified using molecular cloning, 454-pyrosequencing and a Luminex ASR assay, all targeting the ITS region	- *Fusarium* is found in 88% and 3% of vegetarian and conventional diet samples respectively. - *Malassezia* is found in 81% and 12% of vegetarians and conventional diet samples respectively. - *Penicillium* is found in 75% and 1% of vegetarians and conventional diet samples respectively. - *Aspergillus* is found in 68% and 6% of vegetarians and conventional diet samples respectively.
Ghannoum et al.^157^	Mycobiome diet	10 healthy participants (6 male; 3 female)	- Daily food intake, weight, BM for 28 days - Fungi from fecal samples were sequenced using ITS regions	- Adherence to the mycobiome diet decreased the abundance of *Candida* species overall by 72.4% - *C.albicans* decreased by 1.42-fold, while *C.tropicalis* was undetected after 4 weeks - Beneficial fungal species increased after 4 weeks: *Galactomyces geotrichum *and* Pichia kluyver* increased by 58.4% and 45.1% respectively
Auchtung et al.^160^	*S. cerevisiae*-free OR *S. cerevisiae* rich	1 healthy male	*- S. cerevisiae*-free diet for 1 week, followed by 1 day of *S. cerevisiae* rich diet - Fungi, including *S. cerevisiae*, were analyzed using ITS and 18S sequencing	*S. cerevisiae* abundance changed from 0.1% on *S.cerevisiae*-free diet to 86% on the *S.cerevisiae* rich diet

FFQ: food frequency questionnaire; HC: high carbohydrate; ITS: internal transcribed spacer; LC: low carbohydrate; SCFA: short chain fatty acids; SG: study group; CG; control group; TID: three times a day; IU: international unit; ICOG: intestinal Candida overgrowth; CFU: colony forming units; SMRT: single molecular real-time.

In 2019, Pareek et al. observed that the stool microbiota of healthy Indian adults contained a higher proportion of *Candida* and *Prevotella* compared to healthy Japanese, possibly due to the higher consumption of dietary plant polysaccharides by Indian adults.^142^ Tian et al. showed that a high-carbohydrate diet increased the abundance of five fungal genera (*Pleurotus, Kazachstania, Auricularia, Paraphaeosphaeria, Ustilaginaceae)* and decreased the *Blumeria* genus. Conversely, a low-carbohydrate diet resulted in notable shifts in the gut mycobiome, with a depletion of *Blumeria, Agaricomycetes, Malassezia, Rhizopus*, and *Penicillium* genera, and an increase in *Ustilaginaceae*.^143^

Multiple studies have demonstrated that *Candida* species contribute to the fermentation of complex carbohydrates into simple sugars, which serve as an energy source for other microbes.^144^ Research in pigs found that differences in dietary carbohydrate content were associated with variable fungal populations and SCFA production. Specifically, pigs on a lower carbohydrate diet had lower levels of acetate, butyrate and total SCFAs, with several fungal genera being positively correlated with these SCFAs, including *Tomentella, Metschnikowia* and *Loreleia* (Table 2).^145^ Therefore, dietary carbohydrate intake likely influences gut fungal populations and their metabolic activities, however, future studies should focus on elucidating the precise mechanism by which different carbohydrates affect the mycobiome.

**Table 2.** Impact of dietary modulation on gut mycobiome in animal models.

Author	Diet	Number of participants	Study design	Microbial Changes
Mims et al.^156^	Mice split evenly into SC or PD	72 mice – both sexes, 18 mice from 1 of 4 vendors	- 8 week protocol - Fungal communities quantified using ITS2 gene sequencing	- Fungal diversity declined with exposure to PD (compared to SC)
Heisel et al.^152^	Mice fed either SC (18% calories from fat) or HF diet (60% calories from fat)	18 (9 mice/group)	- 16 week protocol - Fungal communities quantified using ITS2 sequencing	- Mice fed SC had higher rates of *Alternaria, Saccharomyces, Septoriella*, and *Tilletiopsis* genera compared to mice fed an HF diet - The most abundant taxa in SC was *S.cerevisiae*, and the most abundant taxa in HF was *Candida parapsilosis*
Li et al.^145^	3 pig breeds fed a corn-soybean diet (free of antibiotics, growth promoters, fungal growth promoters or additives)	30 pigs (10 each from 3 breeds: Chenghua, Yorkshire, and Tibetan)	- Fecal sample consistencies were visually assessed using the subjective score on a 5-point scale (1 = hard feces, 2 = firm well-formed, 3 = soft and partially formed, 4 = loose, semi-liquid, 5 = watery) - Fungal rRNA amplified via PCR in the ITS1 region and DNA sequenced using Illumina HiSeq 2500 platform	- Tormentella positively correlates with the concentration of acetate (*p* < 0.01), butyrate and SCFA (*p* < 0.05) - *Metchinikowia* positively correlates with the concentrations of all SCFAs (acetate, propionate, butyrate, and TSCFAs; *p* < 0.05) - *Loreleia* positively correlates with the concentration of propionate (*p* < 0.05)

HF: high fat; ITS: internal transcribed spacer; PD: processed diet; SC: standard chow.

### Protein-rich diets

The gut microbiota is highly involved in proteolytic fermentation, producing SCFA, branched-chain fatty acids (BCFA), gas, and harmful putrefactive metabolites such as ammonia, amines and hydrogen sulfides.^146^

An in vitro study found that amino acid utilization was positively correlated with fungal growth. The study suggested that amino acids may function as a carbon source for *C.albicans*, as this fungal species produces proteases and has oligopeptide transporters, allowing *C.albicans* to adapt to varying environments through metabolic pathway regulation.^147^ In a separate study, it was found that *S.cerevisiae* and *Aspergillus nidulans* use transporters such as the Amino acid-polyamine-organoCation (APC) superfamily and Major Facilitator Superfamily (MFS) to harness amino acids, which could enhance fungal survival in the gut.^148^

There are limited studies that have assessed the impact of protein-rich diets in humans. However, Shuai et al. examined the factors influencing gut mycobiome homeostasis and found that dairy consumption, a high-protein food source, was negatively associated with *Candida* and positively associated with *Saccharomyces* (Table 1).^149^

The gut microbiota’s role in proteolytic fermentation and the interaction between amino acids and fungal growth highlight the dynamics of the gut mycobiome. Future research should focus on understanding the impact of different protein sources on fungal communities in the gut and exploring dietary interventions to promote a balanced gut mycobiome for improved health outcomes.

### High-fat diets

Dietary fats are digested and absorbed in the small intestine, where they are metabolized to provide the body with energy and various fatty acids. Only one study in humans, undertaken by Hoffman et al., has demonstrated that fat impacts the gut mycobiome. Specifically, they demonstrated that *Candida* was negatively associated with total saturated fat consumption (Table 1).^139^ In vitro studies, have showed that the efficiency in metabolizing fatty acids and fatty alcohols into carbon and energy sources varies greatly among different fungal species.^150^ Research has demonstrated that fatty acids may suppress the growth of fungi. In studies focused on food preservation, saturated fatty acids, unsaturated fatty acids, and oxylipids were found to have antifungal properties.^151^ Due to the lack of studies investigating the effects of fats on the human gut mycobiome, the impact of these dietary components on the gut mycobiome remains unclear. However, in a murine study Heisel et al. showed that mice fed a high-fat diet had increases in six fungal taxa, including *Alternaria, Saccharomyces, Septoriella and Tilletiopsis* genera, compared to mice fed standard chow (Table 2).^152^

While dietary fats are crucial for energy metabolism, their impact on the gut mycobiome is not well understood. Future research is also needed to delve in to the role dietary fats have in shaping the gut mycobiome.

### Dietary habits and patterns

Certain dietary habit and patterns have been found to significantly impact the composition of the gut mycobiome. Sun et al. found that variations in urbanization and dietary habits can lead to differences in the fungal composition of populations living in distinct regions within China (Table 1).^153^ This study identified correlations between the consumption of buttermilk and blueberry tea with the presence of *Tetrapissipora blattae, Sugiyamaella lignohabitans, Kazachstania naganishii*, among others. Moreover, the consumption of black tea and pork was associated with the composition of four species from the *Fusarium* genus.^153^ In a separate study by Ukhanova et al., the ingestion of almonds and pistachios was found to be negatively associated with both *Candida* and *Penicillium*.^154^ Additionally, David et al. performed a comparative analysis between an animal-based and plant-based diet, showing a higher abundance of *Neosartorya fischeri*, a fungus derived from the *Penicillium* genus, in the animal-based diet arm.^155^ Interestingly, studies have shown foodborne fungi, such as *Penicillium* and *Aspergillus*, and potentially pathogenic fungi, such as *Fusarium* and *Malassezia*, in more than 60% of stool samples from vegetarians, but these fungal taxa were infrequently found in individuals who consumed a Western diet.^44,50^ Both populations exhibited high proportions of *Candida* (68% in vegetarian diet samples and 84% in Western diet samples). Mice studies further support the effect of diet modulation on mycobiome profiles. Mims et al. showed that overall fungal diversity decreased in mice fed a processed diet compared to standard chow (Table 2).^156^

Furthermore, recent nutritional studies have utilized the Mycobiome diet to selectively alter the composition of the fungi in the gut. The Mycobiome diet is characterized by the consumption of lean or plant-based protein, mono- or polyunsaturated fats, fiber- and resistant starch-rich foods, and whole foods while avoiding processed, high-sugar foods. Ghannoum et al. demonstrated that following the Mycobiome diet for 28 days led to a 72.4% decrease in the abundance of *Candida*, particularly *C.albicans* and *C.tropicalis*, which decreased by 142% or became undetectable, respectively. Additionally, the Mycobiome diet increased concentrations of some beneficial fungal species, including *Pichia kluyveri* and *Galactomyces geotrichum*. In addition to the beneficial effects this diet had on the gut mycobiome, the diet led to a reduction in bacterial overgrowth in patients with small intestinal bacterial overgrowth, and improved fatigue, sleep, bloating, constipation, diarrhea, and gas in all participants.^157^

An additional noteworthy aspect is the prevalence of common gut fungi such as *S.cerevisiae* and *Debaryomyces hansenii*, in our food supply. These fungi are commonly found in dairy products, bread, fruit skins (including grapes), kombucha and cheeses which makes it difficult to determine if these fungal species truly reside in the gut or are more transient in nature.^116,158,159^ Auchtung et al. revealed that consuming a diet devoid of *S.cerevisiae* led to a large reduction in *S.cerevisiae* levels from 86% at baseline to 0.1% of ITS reads 2 days in to a *S.cerevisiae*-free diet. This suggests that abundance of *S.cerevisiae* is highly dependent on dietary sources of this fungal species.^160^ Consequently, studies exploring the potential impact specific fungal species have in disease should consider the potentially transient nature of food-derived fungal taxa. This consideration is important as it could influence the perceived importance of these species in both health and disease.

It is plausible that the influence of diet on altering the composition of the mycobiome may not be directly attributed to the effects of dietary components on gut fungi. Instead, these effects could be indirect, stemming from the modulation of gut bacteria, which, in turn influences fungal compositions. Due to limited evidence elucidating the mechanisms behind how diet modulates the gut mycobiome, further studies are necessary to discern both the direct and indirect effects of diet on the gut mycobiome.

## Fungal fermentation of dietary components

Despite studies demonstrating that the mycobiome changes in response to diet, our knowledge on the potential impact of diet on the functional capacities of these microorganisms remains limited. It is understood that both bacterial and fungal communities produce metabolites that not only shape their own populations but also exert an influence on other community members and various host cell populations. In response to a high-fiber diet, gut-residing bacteria upregulate functional pathways and enzymes, enabling the fermentation of fibers to produce SCFA, particularly butyrate.^161^ Whether gut fungi share similar metabolic pathways is yet to be elucidated.

Fungi, being eukaryotes, exhibit metabolic distinctions from prokaryotic organisms like bacteria. This underscores the importance of understanding the specific metabolic pathways involved in fungal fermentation of fibers and other dietary components. Select bacteria are known to the produce SCFA acetate, propionate, and butyrate through fiber fermentation. Plant fungi possess metabolic pathways and enzymes to ferment plant cell walls, which predominately comprise of fibers.^162^ However, whether fungi in the gut share these metabolic processes remains uncertain. Some fungal species are employed in the food industry for ethanol production and generating lactic acid and gases via fermentation of fibers such as pectin, cellulose and lignin.^163^ Nevertheless, the specific metabolites produced by gut fungi during dietary fiber fermentation remains uncertain. Furthermore, a recent study focusing on the gut microbiome of goats revealed that fungi outperformed bacteria in degrading cellulose fiber. Fungi produced higher amounts of methane, acetate and formate, while bacteria produced more butyrate and propionate.^117^ Moreover, it is important to consider the role of metabolites in mediating interactions between bacteria and fungi in the gut. Metabolites, such as bile acids, may influence microbial interactions as fungi have the capacity to induce adverse effects on bacterial viability through the transformation of bile acids.^164^

## Conclusion

**Figure 1.**
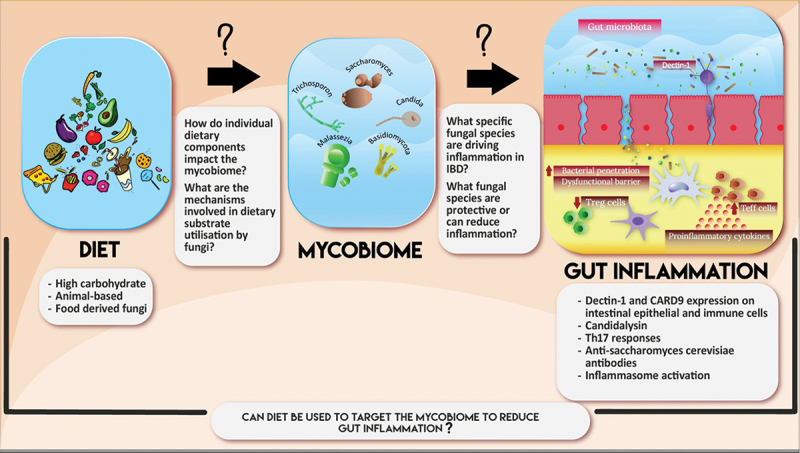
Schematic highlighting what we do and don’t know about the interplay between the diet, the gut mycobiome, and inflammation.

The gut microbiome is a complex, unique entity responsive to changes in environment, diet, and disease. It is teeming with microorganisms from all kingdoms, interacting through complex and dynamic relationships. Fungi play a crucial role in microbiome homeostasis, involved in the regulation of opportunistic pathogens and fermentation of otherwise inaccessible nutrients. Alterations in the mycobiome architecture is associated with dysbiosis, an inflamed immune state implicated in various chronic diseases including IBD. The mycobiome profile varies based on IBD phenotype and severity, with serologic markers showing promise as indicators of disease progression. The impact of diet on the gut mycobiome remains unclear, particularly regarding whether fungi within the GI tract possess the necessary metabolic pathways and enzymes for fermenting various dietary components. Further research is essential to refine fungal DNA extraction protocols and develop databases specific to fungi sequencing. Additionally, there is a need for studies investigating the role of diet in modulating the microbiota beyond bacteria. Mechanistic studies aimed at understanding the metabolic pathways and enzymes involved in fungal fermentation/utilization of dietary components are also crucial. A more profound understanding of how diet shapes the composition and functional capacity of the gut mycobiome is necessary. This knowledge will help determine whether diet-based therapies should specifically target gut fungi, potentially reducing inflammatory processes and improving outcomes for IBD patients (Figure 1).

Several studies have demonstrated that various diets impact the composition of the gut mycobiome. Other studies have additionally shown that a dysbiotic mycobiome can drive inflammation through various mechanisms and is associated with IBD. There are still many unanswered questions linking diet to the mycobiome and GI inflammation. By answering these questions, we will be one step closer to determining whether diet can be used to target the mycobiome to reduce gut inflammation, improving outcomes for IBD patients.

## Appendix A Search Strategy

The following strategy was used to search MEDLINE and EMBASE databases:

[(microbiome/or microbi*) OR (mycobiome/or mycobi* or fungal community/) OR (dysbiosis/) OR (gut health.mp) AND (high fiber diet/) OR (low fiber diet/) OR (cholesterol diet/) OR (obesogenic diet/) OR (chow diet/) OR (fiber free diet/) OR (short chain fatty acid/) OR (fiber/)
